# It Is Time to Feel Better: How Temporal Information of Placebo Analgesia Affects Our Brain

**DOI:** 10.1002/ejp.70093

**Published:** 2025-08-01

**Authors:** Valeria Volpino, Alessandro Piedimonte, Francesco Campaci, Eleonora Maria Camerone, Francesca Persiani, Elisa Carlino

**Affiliations:** ^1^ “Rita Levi Montalcini” Department of Neurosciences University of Turin Turin Italy; ^2^ Carlo Molo Foundation Turin Italy; ^3^ Department of Clinical Neuroscience, Nuffield University of Oxford Oxford UK

## Abstract

**Background:**

Placebo and nocebo effects have been thoroughly studied during the last decades using pain models. Two characteristics have been investigated, namely the direction of the effects (i.e., placebo, amelioration of symptoms/nocebo, worsening of symptoms) and their magnitude (i.e., the robustness of the effects). Here, we propose an investigation of the placebo effects considering a third characteristic: time. We employed functional near‐infrared spectroscopy (fNIRS), an emerging neuroimaging technique suitable for long‐term monitoring and ecological experimental paradigms, to investigate cerebral cortices' activity through oxy‐haemoglobin (O_2_Hb).

**Method:**

42 healthy volunteers were randomised into three groups (No Expectations—NE, Placebo 5′—P5 and Placebo 20′—P20), placebo groups received different information on the timing of a cream's effectiveness (i.e., “the cream will work in 5/20 min”), while the NE group was said they were receiving an inert cream.

**Results:**

Behavioural results showed that pain perception fluctuations mimicked verbal suggestions on cream effectiveness onset. Exploratory analyses of fNIRS signals seem to follow the same pattern: O_2_Hb levels varied by group and time course. In the NE group, no significant differences emerged. In the P5 group, frontal areas were engaged when placebo analgesia occurred soon after treatment, while later, both P5 and P20 showed sustained placebo‐related activations alongside areas linked to time perception and memory.

**Conclusion:**

This study proposes that the cortical network related to the placebo effect may be active and modulated by temporal information of cream effectiveness, as well as their behavioural respective.

**Significance Statement:**

Implementing fNIRS technology, this study confirms previous behavioral findings and begins to show that cerebral networks respond and encode the temporal characteristics of placebo analgesia. Understanding whether the placebo effect can be switched on and off at specific time points through verbal suggestion could be harnessed when clinically beneficial, aligning its timing with that of pharmacological action, especially for drugs with delayed onset, to ensure continuous pain relief, reduce drug intake, and enhance patient comfort.

## 1 Introduction

The placebo effect refers to the amelioration of a symptom due to the administration of an inert treatment, believed to be effective, in a positive psychosocial context (Carlino and Vase 2018; Hohenschurz‐Schmidt et al. 2023). The opposite effect is known as the nocebo effect, which manifests as the worsening of symptoms due to negative expectations (Rossettini et al. 2023). Recent decades have witnessed a surge in scientific interest surrounding these phenomena, prompting investigations into their impact on various domains such as pain (Carlino, Piedimonte, et al. 2016; Carlino, Guerra, et al. 2016; Piedimonte et al. 2020, 2021), motor performance (Beedie et al. 2007; Carlino et al. 2014; Fiorio 2018), visual perception (Piedimonte et al. 2024), gastrointestinal function (Hall et al. 2012), immune response (Prossin et al. 2022), Parkinson's and Alzheimer's diseases (Carlino, Piedimonte, et al. 2016; Matthiesen et al. 2024), depression (Kirsch 2019) and anxiety (Corsi and Colloca 2017).

In the field of pain, experiments have been conducted to examine how expectations can alter pain perception in healthy and patients populations (Rossettini et al. 2022). Two dimensions have been explored: the direction of the effects (i.e., reduction of pain after placebo administration or increase of pain after nocebo administration) and their magnitude (i.e., how robust these effects can be). When analgesia or hyperalgesia is expected, functional magnetic resonance imaging (fMRI) studies have identified the activation of a constellation of brain regions, which involves lateral and medial prefrontal regions, the insula, the somatosensory cortex, as well as the thalamus and the brainstem (Crawford et al. 2021; Wagner et al. 2020). Other techniques, such as electroencephalography (EEG), have also delineated the differential effects of placebo hypoalgesia and nocebo hyperalgesia (Piedimonte et al. 2020). Recent behavioural studies have introduced a third dimension—time—investigating how precise information about the onset of a treatment's effect influences pain perception (Carlino, Piedimonte, et al. 2016; Camerone, Wiech, et al. 2021). Notably, these investigations have demonstrated that information regarding treatment onset can modulate pain in both placebo and nocebo directions. Similar results have been confirmed in the following experiments using different pain models (Camerone et al. 2022; Camerone, Wiech, et al. 2021). Despite these behavioural insights, there remains a dearth of knowledge regarding the underlying neuronal activity. In recent years, functional Near‐Infrared Spectroscopy (fNIRS) has emerged as a non‐invasive technique to investigate changes in blood oxygen levels related to brain activity, primarily referred to as oxygenated haemoglobin (O_2_Hb) and deoxygenated haemoglobin (HHb). While lacking the spatial resolution of fMRI, fNIRS presents advantages, including safety, absence of exclusion criteria, hence applicability in various conditions such as schizophrenia, ADHD, addiction, epilepsy, depression, and autism (Rahman et al. 2020). Unlike fMRI, fNIRS is silent, participant‐friendly, and suitable for conducting long‐term monitoring (Pinti et al. 2018, 2020).

Motivated by these considerations and based on previous consistent results of the employed study design, we aimed to record brain activity with fNIRS to explore pain perception modulation at different time points after the administration of a placebo analgesic cream.

## 2 Materials and Methods

### 2.1 Participants

Computing power analysis with G*Power 3.1 (Faul et al. 2007), 36 participants were needed to achieve a medium effect size (*f* = 0.25; *α* = 0.05; powe*r* = 0.8; *r* = 0.5; *∈* = 1), from (Brączyk and Bąbel 2024; Piedimonte et al. 2024; Valero et al. 2024; Villa‐Sánchez et al. 2019). To prevent dropouts and technical difficulties from impacting our sample size, we recruited 42 healthy volunteers (mean age = 24, sd = 2.74; BMI = 23.74, sd = 4.38; *f* = 21; *m* = 21). They were recruited via group chats and flyers and were informed about the possibility of participating in a study investigating the variations of cerebral activity with fNIRS, following electrical painful stimulation. Participants had no history of chronic pain, and psychiatric diagnosis. Additionally, they were asked not to participate if they were under medications (anxiolytic, antidepressants, and painkillers) and to refrain from consuming alcohol or caffeine for at least 12 h before the experiment. Participants agreed to fulfil a written informed consent before starting the experiment and were informed they could decide to withdraw their consent and data anytime. Submitting the consent, they acknowledged the possibility that certain aspects of the experimental procedure would be intentionally left undisclosed during the experiment and that they would be debriefed at the end for further details; this procedure is known as authorised deception (Miller et al. 2005). Volunteers did not receive any reward for taking part in this study, such as payments or university credits, but were informed they could ask for results once the experiment ended. Data collection started in March 2023 and ended in July 2023. The experimental procedure was conducted according to the policies and ethical principles of the Declaration of Helsinki and approved by the Ethical Committee of the University of Turin (registration number: 0420694).

### 2.2 Group Assignment

Participants were randomised into one of three experimental groups: no expectancy group (NE) and two placebo groups (P5 and P20). Participants in the placebo groups were given positive verbal suggestions as they were informed that an analgesic cream would be applied to their forearms to reduce the perceived painful sensation induced by electrical stimulation. One group was led to believe that the cream would take on its effect after 5 min from the administration (P5 group; *n* = 14) to resemble the effectiveness of a fast‐acting analgesic. The other group was led to believe that the cream would take on its effect after 20 min from the administration (P20 group; *n* = 14), to mimic the effect of an analgesic with a longer onset. Participants assigned to the no expectancies group (NE group; *n* = 14) were given neutral verbal suggestions on the cream without information about temporal parameters. They were informed that an inert cream would be applied to their forearm and that the cream would not influence their perceived painful sensation induced by the electrical stimulation.

### 2.3 Experimental Procedure

**FIGURE 1:**
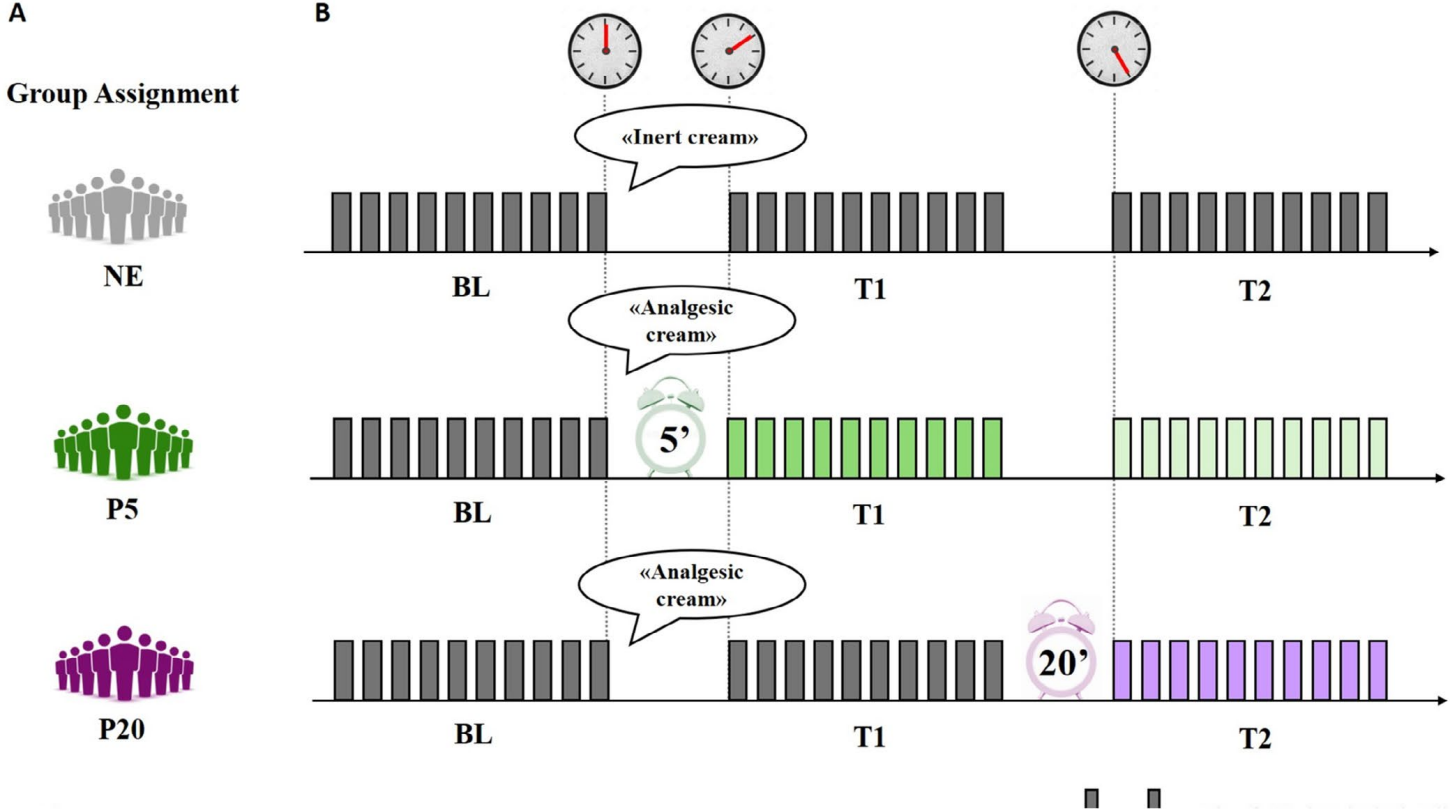
Experiment overview. (A) Group assignments: NE, No Expectations; P5, Placebo 5′; P20, Placebo 20′; (B) Noxious stimulation.

Group assignment and the experimental procedure are outlined in Figure 1. Participants entered the laboratory and filled out the written informed consent and a Qualtrics form on their cellphones about demographics. The experimenter explained the rationale behind the study, leaving out some information to be debriefed at the end of the procedure. Afterward, participants were asked to position their right arm upright on the desk in front of them to facilitate the application of two electrodes on their right forearm, and the assessment of their discomfort threshold began. They were then familiarised with the experimental procedure in a practice run. The practice run consisted of three presentations of a stimulus array, of which participants were asked to rate their perceived pain intensity (from 0 to 10). After the familiarisation, fNIRS was positioned on the subjects' heads, and after a 2 min break, the actual experiment started with the Baseline session. A customised analogic clock with 5 min intervals (i.e., 5–55) was positioned in front of the desk. The clock face also showed an icon of a cream tube at the 12 o'clock position to indicate the time point at which the cream was administered. Participants were informed that the clock was used to help them keep track of time and to know when the next test session was imminent. Subsequently, after the Baseline session, participants were informed about the group they had been assigned to, and the cream was applied in all groups; the experimenter placed a clock hand on the cream icon, indicating the time of the cream administration (Time 0′) and delivered the verbal suggestions. Then, the experimenter exited the room and left some books, giving participants the opportunity to read while waiting for the next test session. Ten minutes after cream administration, the experimenter returned to the laboratory room, and the first test session was run (Test 10′). The run lasted 5 min and was followed by a 10 min rest period, during which the experimenter exited the room again. The second test session was completed 25 min after the cream administration (Test 25′).

### 2.4 Verbal Suggestions About Cream Effectiveness

Verbal suggestions were delivered at the cream administration time (after the Baseline session). A sham cream was administered in all groups; it consisted of AquaGel Solution (2 g) and water (17 mL) and was presented to participants in a clear plastic tube. The cream was transparent, odourless, and tasteless. The experimenter applied the cream within a radius of 2 cm around the electrode and massaged it into the skin for 40 s to ensure that it was fully absorbed.

Placebo groups expected to receive a cream that would decrease their perceived painful sensation. Information to the P5 group was provided as follows: “You have been assigned to the group receiving the analgesic cream, which reduces your perception of pain sensitivity. We are using this cream because its effectiveness has already been demonstrated, meaning it has been shown to reduce the perception of pain. We know that this cream takes effect in 5 min. So, now that it has been applied, we will wait for 5 min for it to function. After 10 min from the application, we will perform a testing procedure (the experimenter indicates 10′ on the clock—Test 1), identical to the one we did earlier. We will repeat the same test again after 25 min from the cream application (the experimenter indicates 25′ on the clock—Test 2). Since we know the cream will take effect in 5 min, in both pain tests (the experimenter indicates 10′ and 25′ on the clock), you will be under the analgesic effect of the cream. What matters to us is comparing brain activity while reducing pain during the 10 and 25 min tests compared to the baseline in which we did not reduce pain”.

Verbal suggestions provided to the P20 group were similar, with the only change of the timing information, set at 20 min: “You have been assigned to the group receiving the analgesic cream, which reduces your perception of pain sensitivity. We are using this cream because its effectiveness has already been demonstrated, meaning it has been shown to reduce the perception of pain. We know that this cream takes effect in 20 min. Now, we will wait a few minutes, and then we will repeat the pain test after 10 min from the application (the experimenter indicates 10′ on the clock—Test 1) and again after 25 min from the cream application (the experimenter indicates 25′ on the clock—Test 2). Since we know the cream will take effect in 20 min, during the first test we will conduct, you will not be under the analgesic effect of the cream. However, during the test we will perform in 25 min, you will be under the analgesic effect of the cream. What interests us is comparing brain activity while reducing pain during the 25 min test compared to when we do not reduce pain in the 10 min test and the baseline test we have already conducted.”

The no‐expectancies group was given information as follows: “You have been assigned to the group receiving the inert cream, which has no influence on your perception of pain sensitivity. We are using this cream because its hydrating‐only effect has already been seen in previous experiments. So, now that it has been applied, we will wait 10 min from the application and perform the testing procedure (the experimenter indicates 10′ on the clock—Test 1), which we did earlier. We will repeat the same test again after 25 min from the cream application (the experimenter indicates 25′ on the clock—Test 2). You are in the control group, which we are using to compare changes in brain activity between you, who will receive this inert cream, and the other groups receiving a different cream (analgesic).”

### 2.5 Noxious Stimulation

Pain was induced using electrical stimuli delivered by a somatosensory stimulator (Digitimer, DS7A), which was connected to two electrodes, applied to the ventral side of the participants' right forearm. The electrode placement site was calculated by distancing them from the wrist by half the distance between the tip of the middle finger and the wrist line. Firstly, volunteers were tested for their discomfort threshold. The stimulation intensity was calibrated using an ascending staircase method (Cornsweet 1957). The electrical stimuli started being delivered under the somatosensory threshold (beginning of the ascending staircase at 0.5 mA), and increased in steps of 0.5 mA till the stimulation elicited discomfort. To control participants' judgement of the stimuli, they were asked to reply, “I didn't feel anything”, “Touch”, or “Discomfort” to the experimenter's question “Which kind of sensation did you feel?”, depending on what they felt. When the discomfort level was reached a brief pause was conceded, and then the procedure had to be repeated three times. The intensity set on the Digitimer for the whole experiment was the mean of the last three ascending intensities at which the participants said “discomfort”.

After calculating the pain threshold, a familiarisation run was performed. Participants underwent three noxious stimulus arrays at pain threshold intensity. During the actual experiment, participants underwent 10 noxious stimulus arrays for every test: the Baseline session (BL), the first test session after 10 min (T1) from the cream administration, and the second test session after 25 min (T2) from the cream administration. Every noxious stimulation was composed of a train of 25 square electrical pulses and lasted 5 s, with an Inter Stimulus Interval (ISI) of 12 s. Ratings of pain were provided verbally after every noxious train (i.e., after each test, the experimenter had 10 pain ratings), using the Numerical Rating Scale (NRS) where 0 represented no pain, 1 was the beginning of a painful sensation, and 10 was unbearable pain. They were guided by images appearing on a computer screen, crafted with Presentation Software (Version 23.0, Neurobehavioral Systems Inc., Berkeley, CA, www.neurobs.com).

### 2.6 Assessment of Expectations

At the end of the experimental procedure, participants were asked to use their phone again to open a Qualtrics form, where expectations about the efficacy of the cream were assessed. The debriefing was done at the end, providing further details on the procedure (disclosing the placebo) and discussing the real aims of the study. This moment was also crucial for the participants to express doubts or concerns about the whole experience. The question asked on the Qualtrics form was about participants' expectations. The question was asked as follows: “When you received the cream, what was your expectation of its effectiveness? (0 = not effective at all; 10 = extremely effective). If you were in the control group, please respond indicating whether you thought the cream would still modulate your pain perception despite the absence of an active ingredient (0 for “not at all”, 10 for “very much”). This question refers to the period before having experienced the placebo/nocebo/inert treatment”. Participants then filled out questions about their past usage of analgesic creams.

### 2.7 fNIRS Acquisition

**FIGURE 2:**
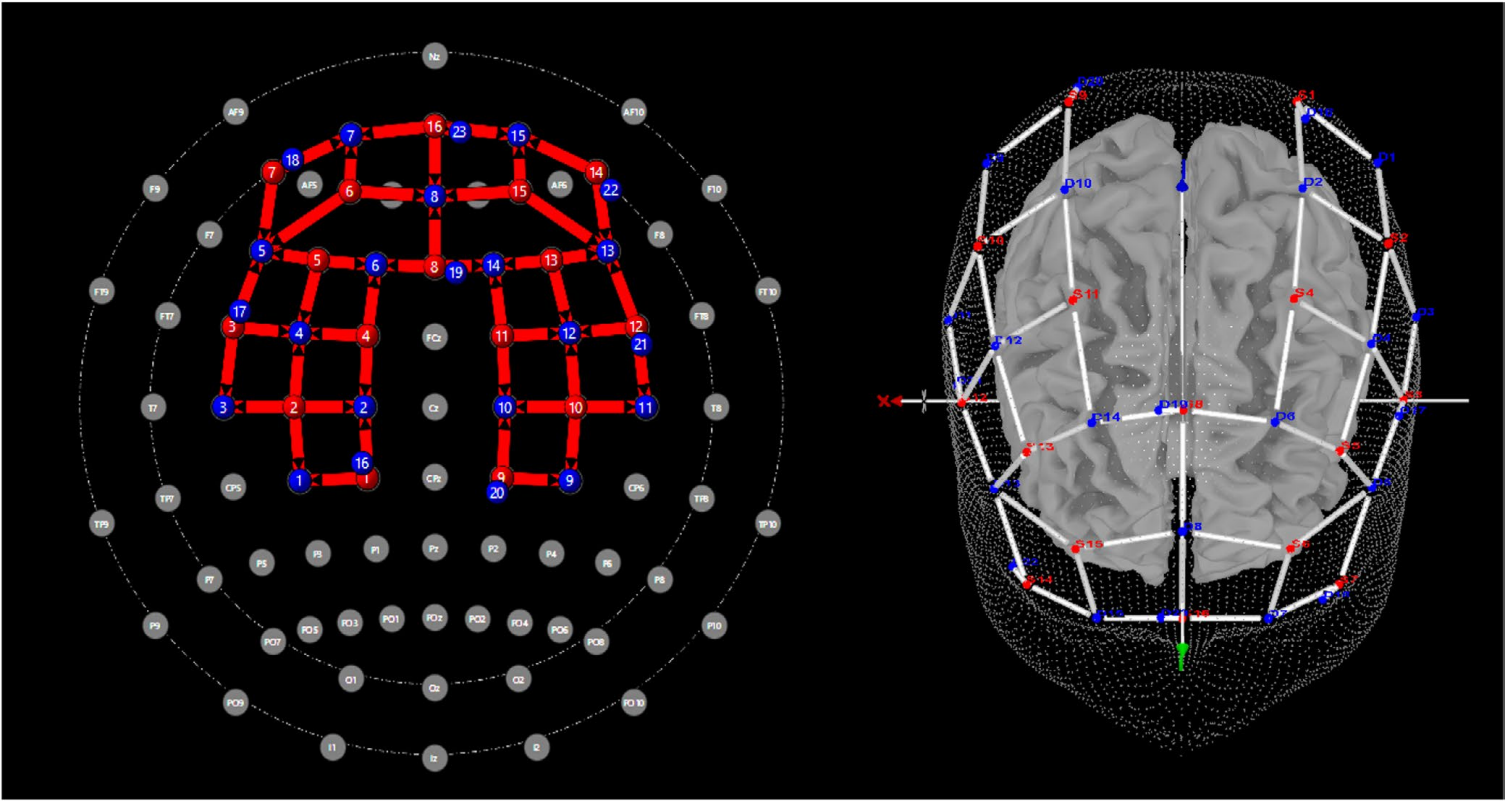
fNIRS optodes positioning following the 10–20 International system and AA2L Atlas from the fOLD toolbox.

Brain hemodynamic signals were recorded using NIRSport 2 (NIRx Medical Technologies LLC, Berlin, Germany; wavelengths of 760 and 850 nm; sampling rate 10.2), which relies on the near‐infrared spectrum light to investigate cerebral blood flow. In total, 16 sources and 16 detectors were mounted on a 32‐channel cap with a source‐detector distance of 30 mm. Moreover, short separation channels (SSC; 15 mm) were added to the cap to measure signals generated by sources other than the cortices activity, such as blood pressure waves, Mayer waves, respiration, and cardiac cycles. Thus, data were recorded from 32 long‐distance channels and 8 short‐distance channels. The fNIRS optodes were positioned based on standard locations calculated using the international 10–20 system. In particular, the positions were defined using the NIRSite program (https://nirx.net/nirsite) and the AAL2 atlas within the fNIRS Optodes Location Decider (fOLD) Matlab toolbox (Zimeo Morais et al. 2018) to cover our region of interests (ROIs): right and left somatosensory cortex (S1), and right and left prefrontal cortex (PFC). fNIRS channels, the corresponding MNI coordinates, and brain regions are outlined in Table 1. The montage representation over the ROIs can be visualised in Figure 2. A signal optimisation check was carried out before starting the recordings to assess the amount of light reaching the cortices. The channels that did not reach acceptable levels of light source intensity (excellent—over 3 mV; acceptable—between 0.5 mV to 3 mV; critical—below 0.5 mV) were fixed by the experimenter. Data were collected at 5.08 Hz sampling frequency.

**TABLE 1:** fNIRS channels positioning using MNI coordinates and corresponding brain areas.

Channels		Sources	Detectors	Coordinates (MNI)			AAL location	MNI location
CH 1	S1‐D1	CP1	CP3	−39	−48	60	Parietal_Inf_L	Left‐VisMotor (7)
CH 2	S1‐D2	CP1	C1	−27	−36	71	Postcentral_L	Left‐SensoryAssoc (5)
CH 4	S2‐D1	C3	CP3	−52	−34	52	Parietal_Inf_L	Left‐PrimSensory (1)
CH 5	S2‐D2	C3	C1	−42	−20	62	Precentral_L	Left‐PrimMotor (4)
CH 6	S2‐D3	C3	C5	−60	−18	37	Postcentral_L	Left‐PrimSensory (1)
CH 7	S2‐D4	C3	FC3	−50	−3	50	Precentral_L	Left‐PreMot+SuppMot (6)
CH 8	S3‐D3	FC5	C5	−62	−3	23	Postcentral_L	Left‐PrimMotor (4)
CH 9	S3‐D4	FC5	FC3	−55	12	34	Precentral_L	Left‐PreMot+SuppMot (6)
CH 10	S3‐D5	FC5	F5	−56	24	20	Frontal_Inf_Tri_L	Left‐Broca‐Triang (45)
CH 12	S4‐D2	FC1	C1	−26	−5	68	Frontal_Sup_2_L	Left‐PreMot+SuppMot (6)
CH 13	S4‐D4	FC1	FC3	−38	12	55	Frontal_Mid_2_L	Left‐PreMot+SuppMot (6)
CH 14	S4‐D6	FC1	F1	−23	26	56	Frontal_Sup_2_L	Left‐FrontEyeFields (8)
CH 15	S5‐D4	F3	FC3	−45	25	41	Frontal_Mid_2_L	Left‐FrontEyeFields (8)
CH 16	S5‐D5	F3	F5	−46	39	26	Frontal_Mid_2_L	Left‐AntPFC (10)
CH 17	S5‐D6	F3	F1	−31	39	41	Frontal_Mid_2_L	Left‐dlPFC (dorsal) (9)
CH 18	S6‐D5	AF3	F5	−39	50	17	Frontal_Mid_2_L	Left‐AntPFC (10)
CH 19	S6‐D7	AF3	Fp1	−24	63	9	Frontal_Sup_2_L	Left‐AntPFC (10)
CH 20	S6‐D8	AF3	Afz	−12	62	23	Frontal_Sup_2_L	Left‐AntPFC (10)
CH 21	S7‐D5	AF7	F5	−47	46	6	Frontal_Mid_2_L	Left‐dlPFC (lat) (46)
CH 22	S7‐D7	AF7	Fp1	−33	59	−2	Frontal_Mid_2_L	Left‐AntPFC (10)
CH 24	S8‐D6	Fz	F1	−9	41	50	Frontal_Sup_Medial_L	Left‐FrontEyeFields (8)
CH 25	S8‐D8	Fz	Afz	2	50	39	Frontal_Sup_Medial_L	dlPFC (dorsal) (9)
CH 26	S8‐D14	Fz	F2	10	41	50	Frontal_Sup_Medial_R	Right‐FrontEyeFields (8)
CH 28	S9‐D9	CP2	CP4	39	−49	60	Parietal_Sup_R	Right‐VisMotor (7)
CH 29	S9‐D10	CP2	C2	28	−36	71	Postcentral_R	Right‐PrimSensory (1)
CH 31	S10‐D9	C4	CP4	53	−35	52	Parietal_Inf_R	Right‐SupramargGyr (40)
CH 32	S10‐D10	C4	C2	42	−21	62	Precentral_R	Right‐PrimMotor (4)
CH 33	S10‐D11	C4	C6	62	−20	37	Postcentral_R	Right‐SupramargGyr (40)
CH 34	S10‐D12	C4	FC4	52	−4	48	Precentral_R	Right‐PreMot+SuppMot (6)
CH 35	S11‐D10	FC2	C2	27	−4	68	Frontal_Sup_2_R	Right‐PreMot+SuppMot (6)
CH 36	S11‐D12	FC2	FC4	39	12	54	Frontal_Mid_2_R	Right‐FrontEyeFields (8)
CH 37	S11‐D14	FC2	F2	24	26	55	Frontal_Sup_2_R	Right‐FrontEyeFields (8)
CH 38	S12‐D11	FC6	C6	64	−5	22	Postcentral_R	Right‐PrimMotor (4)
CH 39	S12‐D12	FC6	FC4	56	12	33	Precentral_R	Right‐Broca‐Operc (44)
CH 40	S12‐D13	FC6	F6	58	24	18	Frontal_Inf_Tri_R	Right‐dlPFC (dorsal) (9)
CH 42	S13‐D12	F4	FC4	44	25	40	Frontal_Mid_2_R	Right‐FrontEyeFields (8)
CH 43	S13‐D13	F4	F6	46	38	24	Frontal_Mid_2_R	Right‐dlPFC (dorsal) (9)
CH 44	S13‐D14	F4	F2	30	40	41	Frontal_Mid_2_R	Right‐dlPFC (dorsal) (9)
CH 45	S14‐D13	AF8	F6	48	46	5	Frontal_Mid_2_R	Right‐dlPFC (lat) (46)
CH 46	S14‐D15	AF8	Fp2	34	59	−2	Frontal_Mid_R	Right‐AntPFC (10)
CH 48	S15‐D8	AF4	Afz	13	61	24	Frontal_Sup_2_R	Right‐AntPFC (10)
CH 49	S15‐D13	AF4	F6	40	50	16	Frontal_Mid_2_R	Right‐AntPFC (10)
CH 50	S15‐D15	AF4	Fp2	25	63	9	Frontal_Sup_2_R	Right‐AntPFC (10)
CH 51	S16‐D7	Fpz	Fp1	−12	67	0	Frontal_Sup_2_L	Left‐AntPFC (10)
CH 52	S16‐D8	Fpz	AFz	1	64	14	Frontal_Sup_Medial_L	AntPFC (10)
CH 53	S16‐D15	Fpz	Fp2	13	67	0	Frontal_Sup_2_R	Right‐AntPFC (10)

*Note:* For each Channel, AAL and MNI locations are reported.

Abbreviations: AntPFC, Anterior PreFrontal Cortex; Broca‐Operc, Broca Operculum; dlPFC, dorso‐lateral PreFrontal Cortex; PrimMotor, Primary Motor Cortex; Sensory Assoc, Sensory Associative Cortex; SupramargGyr, Supramarginal Gyrus.

### 2.8 fNIRS Pre‐Processing

fNIRS data were analysed using the Satori Software (v.2.0) by Brain Innovation (NIRx Medical Technologies, https://nirx.net/satori). The preprocessing was performed on the whole length of the raw data for each participant following a standard approach for fNIRS analysis (Yücel et al. 2021). Data were first converted into optical density (OD), and then motion correction was applied to exclude any artefacts derived from participants' movement. Motion correction functions of Satori were used, in particular, Spike Removal OD and Temporal Derivative Distribution Repair (TDDR) OD (Fishburn et al. 2019). Temporal filtering was computed as follows to account for cardiac oscillations and Mayer waves: 0.5 Hz Butterworth low‐pass filter, 0.01 Hz Butterworth high‐pass filter, and the linear detrending function of Satori. Data were also regressed to the highest correlates short‐separation channel (SSD) to improve the quality of the hemodynamic response function (HRF) (Yücel et al. 2015). After computing the first steps of pre‐processing, the data were converted from optical density to concentration changes (CC) in the absorption of near‐infrared light of oxygenated‐haemoglobin (O_2_Hb) and deoxygenated‐haemoglobin (HHb) through the modified Beer–Lambert Law (mBLL). CC data were z‐transformed and trimmed 10s before the first event and 20s after the last event to leave out from the GLM estimation the signal not related to the task.

## 3 Statistical Analysis

The Kolmogorov–Smirnov test was used to verify the normality of the distribution of all the dependent variables. In no case was the normality violated. Analyses were performed using Statistica Software (version 10) (StatSoft 2011), JASP Team (2024), JASP (version 0.19.3) [https://jasp‐stats.org/], and IBM SPSS Statistics (version 29.0.2.0).

### 3.1 Behavioural Analysis

Mean pain intensity rating was calculated for each session (i.e., Baseline, Test 1, and Test 2) by averaging across the 10 pain ratings per session, resulting in 3 mean pain intensity values for each participant. To test baseline differences in demographic and psychological variables and in baseline pain intensity scores, we first compared the 3 groups using an analysis of variance (ANOVA). To test the placebo analgesic effect in the different groups at different times, mean pain intensity ratings at Test 1 and Test 2 of the placebo and NE groups were entered into a 3 × 3 mixed ANOVA with the within‐subjects factor TIME (three levels: Baseline vs. Test 1 vs. Test 2) and between‐subjects factor GROUP (three levels: P5 vs. P20 vs. NE). To test differences in the assessment of expectations on the cream effectiveness, we computed a one‐way ANOVA with GROUP as a between‐subject factor (three levels: P5 vs. P20 vs. NE). Significant effects were followed up using a pairwise Student–Newman–Keuls (SNK) post hoc test. The significance level was set at α = 0.05. Additionally, correlation analyses were performed on the expectations rates and the pain percentage differences between Baseline and Test 1 and between Baseline and Test 2: positive values account for a reduction in pain perception, thus, an improvement in participants conditions (placebo effect); negative values account for an increase in pain perception, thus, a worsening in participants conditions. Correlation analysis was also carried out on pain percentage differences and beta values changes between paradigm conditions. Spearman's rho was used to evaluate the correlations between variables, and Benjamini‐Hochberg's procedure was carried out with the adjusted alpha level to control for the false discovery rate (FDR) caused by multiple comparisons (Benjamini and Hochberg 1995). Mediation analysis was also performed with Model 4 of the PROCESS package for SPSS (Hayes 2018). The level of confidence for all confidence intervals was set at 95% and the number of bootstraps sampled for the percentile bootstrap confidence intervals was 5000. The significance level was set at α = 0.05.

### 3.2 fNIRS Analysis

Analyses were performed using Satori Software (v.2.0) by Brain Innovation (NIRx Medical Technologies, https://nirx.net/satori) and Statistica Software (v10) (StatSoft 2011). Beta weights are the output of the GLM approach in Satori. The General Linear Model (GLM) aims to “explain” or “predict” the variation in a dependent variable using a linear combination (weighted sum) of several reference functions. In this context, the dependent variable is the observed fNIRS time course of a channel, and the reference functions represent the *idealised* fNIRS response time courses for different experimental conditions (i.e., an estimation of the time course of O_2_Hb and HHb in NE, P5, and P20 at different time points). The GLM fitting procedure must find a set of beta values explaining the data as well as possible. The beta weight of a condition predictor measures the extent to which its time course contributes to explaining the channel time course. A good fit would be achieved with beta values leading to predicted values. The beta weight of a condition predictor measures the extent to which its time course contributes to explaining the channel time course. In other words, the beta values, or weights, represent the goodness of fit of the GLM model; hence, the higher or lower levels of O_2_Hb and HHb in different conditions (for further insight see Pinti et al. 2017; Plichta et al. 2007).

We computed beta values for each participant, channel, and condition. Our analysis focused on the beta values estimated by a general linear model related to the O_2_Hb. Beta values were statistically analysed by mixed‐model analysis of variance (ANOVA) with the within‐subjects factor TIME (Baseline vs. Test 1 vs. Test 2) and CHANNEL (CH1:CH46), and the between‐subjects factor GROUP (NE vs. P5 vs. P20). The significance level was set at α = 0.05. Benjamini‐Hochberg's procedure was carried out with the adjusted alpha level to control for the false discovery rate (FDR) caused by multiple comparisons (Benjamini and Hochberg 1995).

## 4 Results

### 4.1 Demographic Information

Overall, 42 healthy participants were recruited for the experiment. Participants did not differ in age, sex, and body mass index (BMI).

### 4.2 Mean Pain Intensity Ratings

**FIGURE 3:**
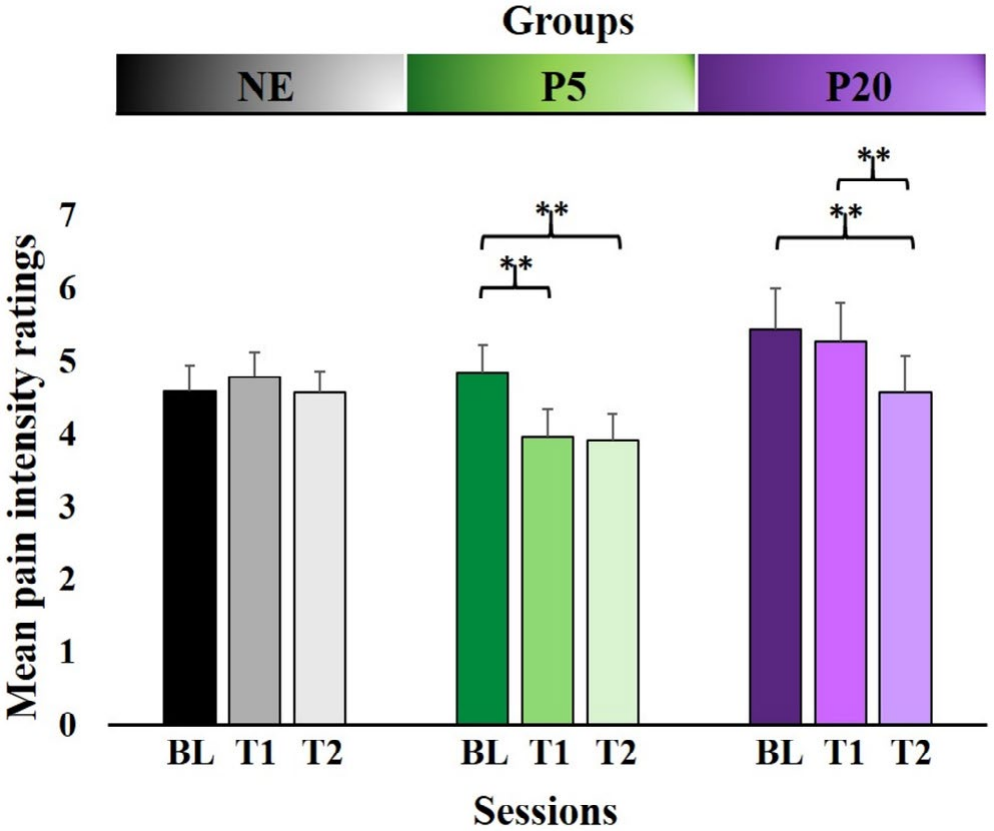
Behavioural results. Upper part: Group assignment—No Expectation group (NE), Placebo 5′ group (P5), and Placebo 20′ group (P20). Lower part: Sessions—BL, Baseline; T1, Test 1; T2, Test 2. On the *y*‐axis are reported mean pain intensity ratings. *p < 0.05; **p < 0.01. Error bars represent standard errors of the means (SEM).

Mean pain intensity ratings are reported in Figure 3. ANOVA showed a significant main effect of TIME [F(2, 78) = 25.2, *p* < 0.001, *η*_p_^2^ = 0.39], and a significant interaction effect of TIME*GROUP [F(4, 78) = 10.27, *p* = < 0.001, *η*_p_^2^ = 0.34]. In the NE group (Baseline—mean = 4.59, sd = 1.27, Test 1—mean = 4.79, sd = 1.20, Test 2—mean = 4.56, sd = 1.08), no significant differences were observed. In the P5 group (Baseline—mean = 4.84, sd = 1.46, Test 1—mean = 3.96, sd = 1.43, Test 2—mean = 3.91, sd = 1.39), the SNK post hoc test showed a significant reduction in mean pain intensity ratings in Test 1 compared with Baseline (*p* < 0.001) as well as in Test 2 compared with Baseline (p < 0.001), but no significant difference between Test 1 and Test 2 (*p* > 0.05). Furthermore, in the P20 group (Baseline—mean = 5.44, sd = 2.10, Test 1—mean = 5.27, sd = 1.96, Test 2—mean = 4.57, sd = 1.87), post hoc SNK results showed a significant reduction in mean pain intensity ratings in Test 2 compared with Baseline (*p* < 0.001) and in Test 1 compared with Test 2 (p < 0.001), but, interestingly, no significant difference between Baseline and Test 1 (*p* > 0.05). These results suggest a significant reduction in mean pain intensity ratings at Tests 1 and 2 in the P5 group, while the same modulation occurs only at Test 2 in the P20 group, supporting our hypothesis that the placebo cream administration actively reduced perceived pain intensity, following the induced expectations on the time course of its effectiveness. To sum up, the P5 group has reduced mean pain intensity ratings both at Tests 1 and 2, after the induced expectations that the cream worked only after 5 min from the administration, meanwhile, the P20 group showed reduced mean pain intensity just at Test 2, after the induced expectations that the cream worked after 20 min from the administration (i.e., only Test 2 was subjected to the placebo effect).

### 4.3 Assessment of Expectations on Cream Effectiveness

The one‐way ANOVA conducted on the expectations rates of cream effectiveness showed a main effect of GROUP [F (3,39) = 5.67, *p* < 0.05, *η* ^2^ = 0.26]. Post hoc SNK test results showed significantly higher expectancy scores for the P5 group (*p* < 0.05) and the P20 group (*p* < 0.05) compared to the NE group. Instead, no difference was found between the P5 group and the P20 group (*p* > 0.05). These results support the strong belief in cream effectiveness held by the two placebo groups, previously suggested with verbal information on perceived pain reduction after cream administration.

### 4.4 Correlation Analysis on Expectations and Differences in Pain Perception

**FIGURE 4:**
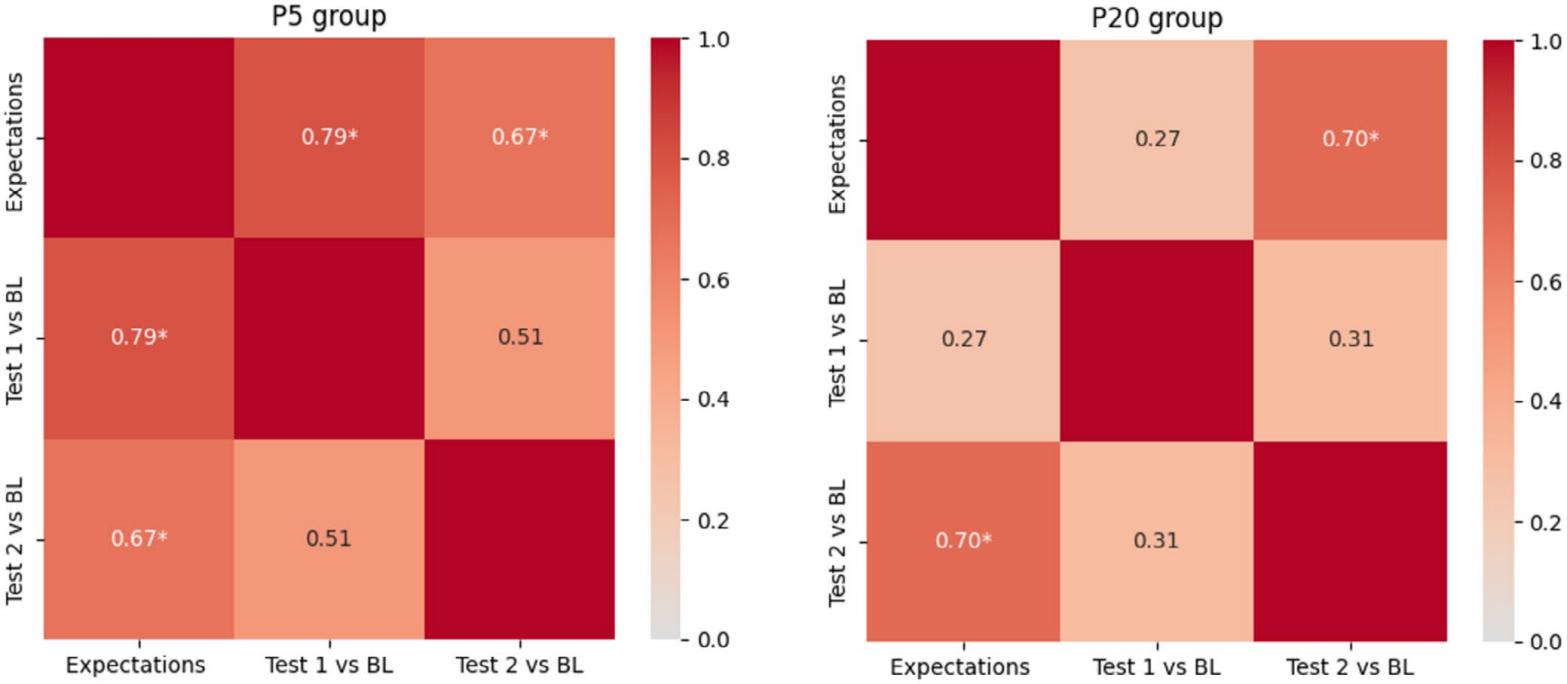
Correlation analysis between expectations and differences in pain perception from Baseline to Test sessions. On the left, the results for the P5 group, on the right, the results from the P20 group. *p < 0.05; **p < 0.01.

Spearman's rho analysis for the P5 group found a strong positive correlation between expectation rates and pain percentage differences at Test 1 (*ρ* = 0.790, *p* = 0.0007) and at Test 2 (*ρ* = 0.667, *p* = 0.009), indicating a statistically significant association. For the P20 group, the analysis found no significant correlation between expectation rates and pain percentage differences at Test 1 (*ρ* = 0.265, *p* = 0.359) but found a strong positive correlation at Test 2 (*ρ* = 0.704, *p* = 0.005). These results suggest that higher expectations were associated with greater reduction in pain perception; thus, greater percentage differences from the Baseline session (previous to placebo effect induction) (Figure 4).

### 4.5 Mediation Analysis

**FIGURE 5:**
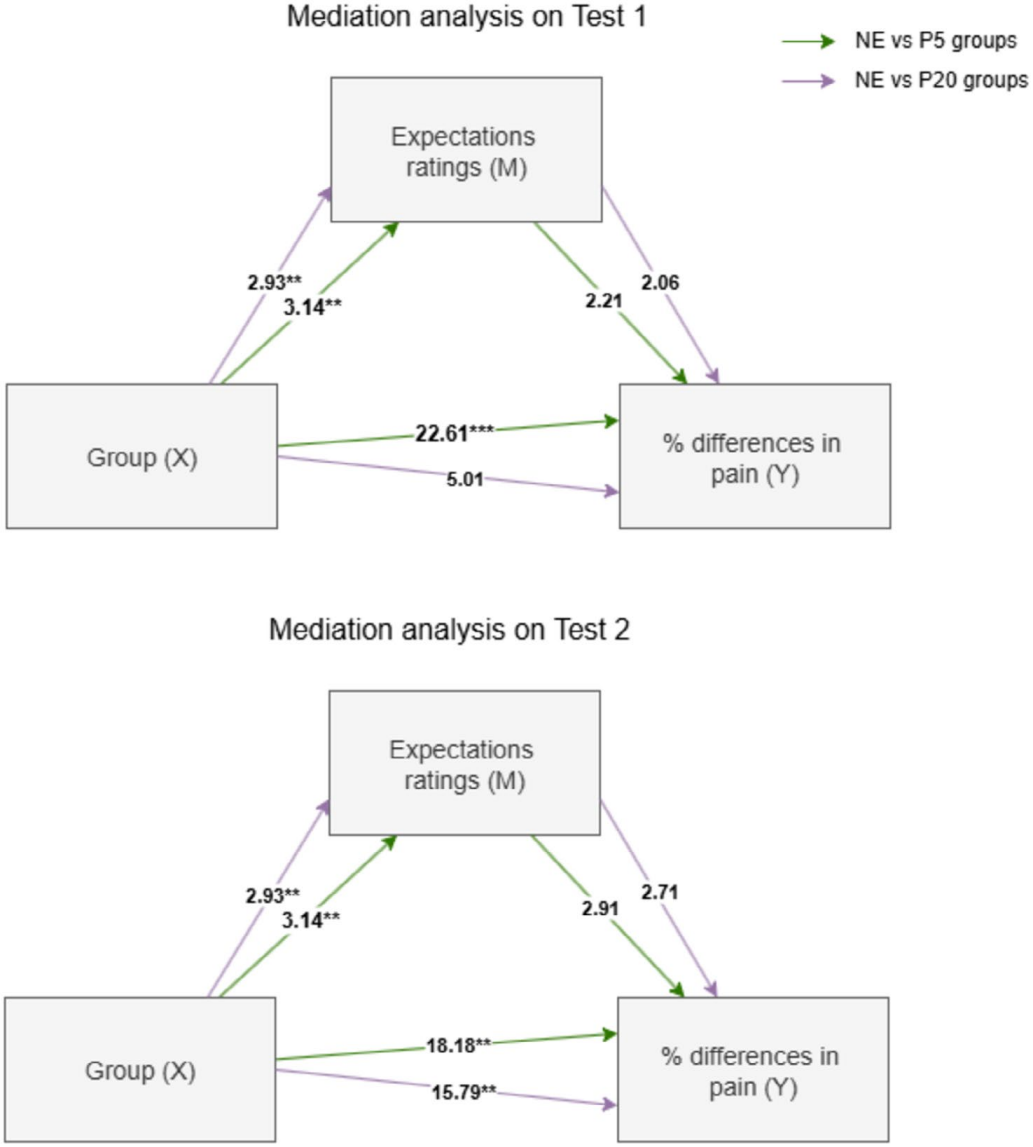
Mediation analysis for expectations and groups on differences in pain perception from Baseline to Test sessions. On top, the results on the differences in Test 1, on the bottom, the results on the differences in Test 2. For both analyses, the inner circle (green) represents the contrast between NE and P5 groups, and the outer circle (purple) represents the contrast between NE and P20 groups. *p < 0.05; **p < 0.01.

To test whether the expectation ratings were effective in mediating the differences in pain perception ratings from the Baseline sessions (previous to placebo effect induction), we introduced a mediation model with X as the independent categorical variable (three group levels: NE, P5 group and P20 group), M as the mediation variable (expectation ratings) and Y as the dependent variable (percentage differences between Test sessions and Baseline). The mediation model was computed for Test 1 and for Test 2 (Figure 5).

The results of the mediation analysis on the Test 1 pain percentage differences revealed a significant direct effect of the group on the expectations ratings (*a*) and on the pain percentage differences (*c*) from Baseline, for the contrast NE versus P5 group (*a*—b = 3.14, t(39) = 3.02, *p* = 0.005, SE = 1.04, 95% CI: 1.03 to 5.25; *c*—b = 22.61, t(38) = 4.07, *p* = 0.0002, SE = 5.56, 95% CI: 11.35 to 33.87) and only on expectations ratings (*a*) for the contrast NE versus P20 (*a*—b = 2.93, t(39) = 2.81, *p* = 0.007, SE = 1.04, 95% CI: 0.82 to 5.04; *c*—b = 5.01, t(38) = 0.91, *p* = 0.368, SE = 5.49, 95% CI: −6.11 to 16.13), supporting previously outlined findings with ANOVAs. No indirect effect (*a* × *b*) of expectations on pain percentage differences (*b*) was found for NE vs. P5 group (*a* × *b =* 2.21, BootSE = 2.88, BootLLCI = −4.48, BootULCI = 7.49) and for the NE versus P20 group (*a* × *b =* 2.06, BootSE = 2.97, BootLLCI = −3.6, BootULCI = 8.48).

The results of the mediation analysis on the Test 2 pain percentage differences revealed a significant direct effect of the group on the expectations ratings (*a*) (the same as the one obtained from the previous mediation analysis) and on the pain percentage differences (*c*) from Baseline for the contrast NE vs. P5 group (*c*—b = 18.18, t(38) = 3.38, *p* = 0.002, SE = 5.39, 95% CI: 7.28 to 29.09) and for the contrast NE versus P20 group (*c* − b = 15.79, t(38) = 2.97, *p* = 0.005, SE = 5.32, 95% CI: 5.026 to 26.56), supporting previously outlined findings with ANOVAs. No indirect effect (*a* × *b*) of expectations on pain percentage differences (*b*) was found for NE versus P5 group (*a* × *b =* 2.91, BootSE = 2.07, BootLLCI = −1.11, BootULCI = 7.23) and for the NE versus P20 group (*a* × *b =* 2.71, BootSE = 2.01, BootLLCI = −1.24, BootULCI = 6.85).

### 4.6 fNIRS Results

**FIGURE 6:**
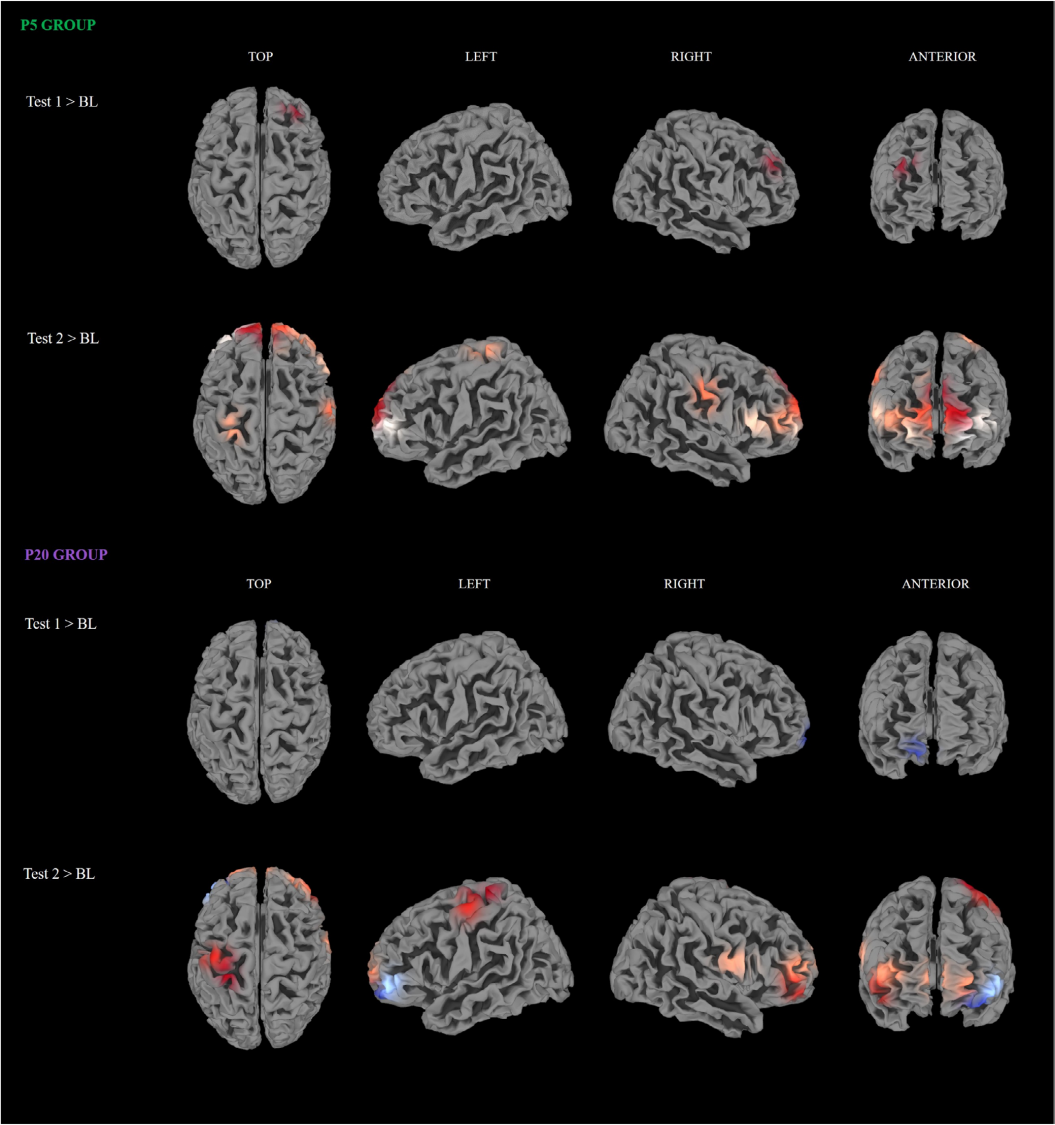
fNIRS results. Brain areas activation in Test 1 and Test 2 compared to Baseline, in P5 and P20 groups. Increases in O_2_Hb levels are displayed in red, while decreases in O_2_Hb levels are displayed in blue.

Brain activations are displayed in Figure 6. ANOVA showed a significant main effect of TIME [F(2, 78) = 18.94, *p* < 0.001, *η*_p_^2^ = 0.33], a significant interaction effect of TIME*CHANNEL [F(90, 3510) = 3.04, *p* < 0.001, *η*_p_^2^ = 0.15], and a significant interaction effect of TIME*CHANNEL*GROUP [F(180, 3510) = 3.33, *p* < 0.001, *η*_p_^2^ = 0.28]. Benjamini‐Hochberg test revealed significant differences in channels related to our ROIs. All significant channels and ROIs, *p*‐values, *q*‐values, and *t*‐values are presented in Table 2.

**TABLE 2:** Post hoc Benjamini‐Hochberg analysis on all channels and conditions.

Channel		MNI locationh	*q*	*p*	*t*
*Contrast P5 Group—Test 10′* vs *Baseline*					
CH 44	S13‐D14	Right‐dlPFC (dorsal) (9)	0.001690821	0.0008252	432.4328
*Contrast P5 Group—Test 25′ > Baseline*					
CH 2	S1‐D2	Left‐SensoryAssoc (5)	0.000785024	0.0002158	50.6736
CH 18	S6‐D5	Left‐AntPFC (10)	0.002958937	0.002591	37.1576
CH 19	S6‐D7	Left‐AntPFC (10)	0.00326087	0.0032308	360.0173
CH 20	S6‐D8	Left‐AntPFC (10)	0.000120773	8264E‐06	708.2329
CH 25	S8‐D8	dlPFC (dorsal) (9)	603865E‐05	7554E‐06	714.3106
CH 26	S8‐D14	Right‐FrontEyeFields (8)	0.000845411	0.0002297	503.1685
CH 33	S10‐D11	Right‐SupramargGyr (40)	0.000543478	8036E‐05	564.2103
CH 40	S12‐D13	Right‐dlPFC (dorsal) (9)	0.00205314	0.000955	424.5575
CH 42	S13‐D12	Right‐FrontEyeFields (8)	0.002898551	0.0024792	−37.3892
CH 48	S15‐D8	Right‐AntPFC (10)	0.000362319	3841E‐05	609.0125
CH 49	S15‐D13	Right‐AntPFC (10)	0.000724638	0.0002133	507.3993
CH 50	S15‐D15	Right‐AntPFC (10)	0.002294686	0.0014044	403.9287
CH 52	S16‐D8	AntPFC (10)	0.000422705	4707E‐05	596.5054
*Contrast P5 Group—Test 25′* vs *Test 10′*					
CH 2	S1‐D2	Left‐SensoryAssoc (5)	0.001147343	0.0003551	478.6863
CH 20	S6‐D8	Left‐AntPFC (10)	0.000181159	1708E‐05	66.0278
CH 25	S8‐D8	dlPFC (dorsal) (9)	0.000301932	2618E‐05	632.9752
CH 26	S8‐D14	Right‐FrontEyeFields (8)	0.001086957	0.0003148	48.5423
CH 32	S10‐D10	Right‐PrimMotor (4)	0.002838164	0.002211	−37.9912
CH 33	S10‐D11	Right‐SupramargGyr (40)	0.000664251	0.0001576	524.7309
CH 40	S12‐D13	Right‐dlPFC (dorsal) (9)	0.0022343	0.001319	407.2676
CH 42	S13‐D12	Right‐FrontEyeFields (8)	0.002173913	0.0013095	−40.7655
CH 43	S13‐D13	Right‐dlPFC (dorsal) (9)	0.002657005	0.0020005	385.1855
CH 48	S15‐D8	Right‐AntPFC (10)	0.000483092	6498E‐05	576.9258
CH 49	S15‐D13	Right‐AntPFC (10)	0.00102657	0.0003028	487.5952
CH 50	S15‐D15	Right‐AntPFC (10)	0.00307971	0.0029779	364.2825
CH 52	S16‐D8	AntPFC (10)	0.000241546	2599E‐05	633.4352
*Contrast P20 Group—Test 10′* vs *Baseline*					
CH 53	S16‐D15	Right‐AntPFC (10)	0.001992754	0.0009388	−42.5479
*Contrast P20 Group—Test 25′* vs *Baseline*					
CH 2	S1‐D2	Left‐SensoryAssoc (5)	0.001328502	0.0004266	468.5042
CH 5	S2‐D2	Left‐PrimMotor (4)	0.001449275	0.0006008	449.6815
CH 19	S6‐D7	Left‐AntPFC (10)	0.002415459	0.0018225	390.1066
CH 21	S7‐D5	Left‐dlPFC (lat) (46)	0.003140097	0.0030732	−36.2632
CH 22	S7‐D7	Left‐AntPFC (10)	0.000905797	0.0002661	−49.4856
CH 38	S12‐D11	Right‐PrimMotor (4)	0.002717391	0.0020516	38.3855
CH 45	S14‐D13	Right‐dlPFC (lat) (46)	0.001388889	0.0005828	451.3408
CH 49	S15‐D13	Right‐AntPFC (10)	0.002596618	0.0019844	385.6116
CH 52	S16‐D8	AntPFC (10)	0.002777778	0.0021091	382.3979
*Contrast P20 Group—Test 25′* vs *Test 10′*					
CH 2	S1‐D2	Left‐SensoryAssoc (5)	0.001751208	0.0008416	431.3729
CH 5	S2‐D2	Left‐PrimMotor (4)	0.001871981	0.0009174	42.6721
CH 22	S7‐D7	Left‐AntPFC (10)	0.001570048	0.0007029	−44.1125
CH 38	S12‐D11	Right‐PrimMotor (4)	0.003019324	0.0029473	364.8218
CH 45	S14‐D13	Right‐dlPFC (lat) (46)	0.001268116	0.0004152	469.9975
CH 49	S15‐D13	Right‐AntPFC (10)	0.002536232	0.0019646	386.1393
CH 52	S16‐D8	AntPFC (10)	0.001509662	0.0006097	448.8724
*Contrast between groups C* vs *P5—Test 25′*					
CH 32	S10‐D10	Right‐PrimMotor (4)	0.001630435	0.0007566	381.4706
CH 36	S11‐D12	Right‐FrontEyeFields (8)	0.002475845	0.0019561	344.3776
CH 39	S12‐D12	Right‐Broca‐Operc (44)	0.003321256	0.0033169	323.3299
*Contrast between groups C* vs *P20—Test 25′*					
CH 22	S7‐D7	Left‐AntPFC (10)	0.002355072	0.0016037	352.2059
CH 45	S14‐D13	Right‐dlPFC (lat) (46)	0.001630435	0.0009269	−3.7361
*Contrast between groups P5* VS *P20—Test 25′*					
CH 5	S2‐D2	Left‐PrimMotor (4)	0.000966184	0.000289	−41.8391
CH 36	S11‐D12	Right‐FrontEyeFields (8)	0.003200483	0.0030775	−32.6338
CH 22	S7‐D7	Left‐AntPFC (10)	0.000603865	8367E‐05	465.4492
CH 40	S12‐D13	Right‐dlPFC (dorsal) (9)	0.001207729	0.0003621	409.7852
CH 33	S10‐D11	Right‐SupramargGyr (40)	0.001811594	0.0009007	374.7204
CH 21	S7‐D5	Left‐dlPFC (lat) (46)	0.002113527	0.001251	361.9363

*Note:* Shown are the brain areas that reached significance in all contrasts computed. MNI locations, *q*‐values, *p*‐values and *t*‐values for all brain areas that reached significance are listed.

In the P5 group, channel 44 (Right‐dlPFC) was significantly higher in Test 1 compared to Baseline; meanwhile, channel 18, 19, and 20 (Left‐AntPFC), channel 48, 49, and 50 (Right‐AntPFC), channel 52 (AntPFC), channel 40 (Right‐dlPFC‐dorsal), channel 25 (dlPFC), channel 33 (Right‐SupramargGyr), and channel 2 (Left‐SensoryAssoc) were significantly higher in Test 2 compared to Baseline. Furthermore, in the P5 group, the contrast between Test 2 and Test 1 evidenced significant increases in the levels of O_2_Hb in channel 20 (Left‐AntPFC), channel 48, 49, and 50 (Right‐AntPFC), channel 52 (AntPFC), channel 40 and 43 (Right‐dlPFC), channel 25 (dlPFC), channel 33 (Right‐SupramargGyr), and channel 2 (Left‐SensoryAssoc). These results show that, in the P5 group, during both sessions (Test 1 and Test 2) compared to Baseline, placebo‐related brain areas are engaged.

In the P20 group, only channel 53 (Right‐AntPFC) showed a significant decrease in O_2_Hb levels in Test 1 compared to Baseline, and no other differences were found. Channels 19 (Left‐AntPFC), channel 49 (Right‐AntPFC), channel 52 (AntPFC), channel 45 (Right‐dlPFC), and channel 2 (Left‐SensoryAssoc) were significantly higher in Test 2 compared to Baseline. Channel 49 (Right‐AntPFC), 52 (AntPFC), 45 (Right‐dlPFC), and 2 (Left‐SensoryAssoc) were significantly higher in the P20 group in Test 2 compared with Test 1.

These results show two crucial points: (1) in the P20 group, when comparing Test 1 to Baseline, areas related to placebo responsiveness are not engaged and there is a decrease in O_2_Hb levels in the Right‐PFC, and (2) placebo effect‐related brain regions are aroused when comparing Test 2 with Test 1, following induced expectations on the placebo treatment.

For the NE group, no significant within‐differences (Baseline vs. Test 1 vs. Test 2) were found in any channel. Between‐groups contrasts (NE vs. P5 vs. P20) were also computed. No significant differences were found at Baseline, and Test 1. Results for the contrasts at Test 2 showed significantly higher levels of O_2_Hb in the NE group compared with the P5 group, in channel 32 (Right‐PrimMotor), channel 36 (Right‐FrontalEyeFields), and channel 39 (Right‐Broca‐Operc), but no significant differences in our ROIs. Contrast between NE and P20 at Test 2 showed a significant increase in O_2_Hb in channel 22 (Left‐AntPFC), and a significant decrease in O_2_Hb in channel 45 (Right‐dlPFC). This is consistent within‐ROIs activation related to time and to placebo responsiveness, outlined in previous paragraphs.

### 4.7 Correlations Between Behavioural and fNIRS Findings

**FIGURE 7:**
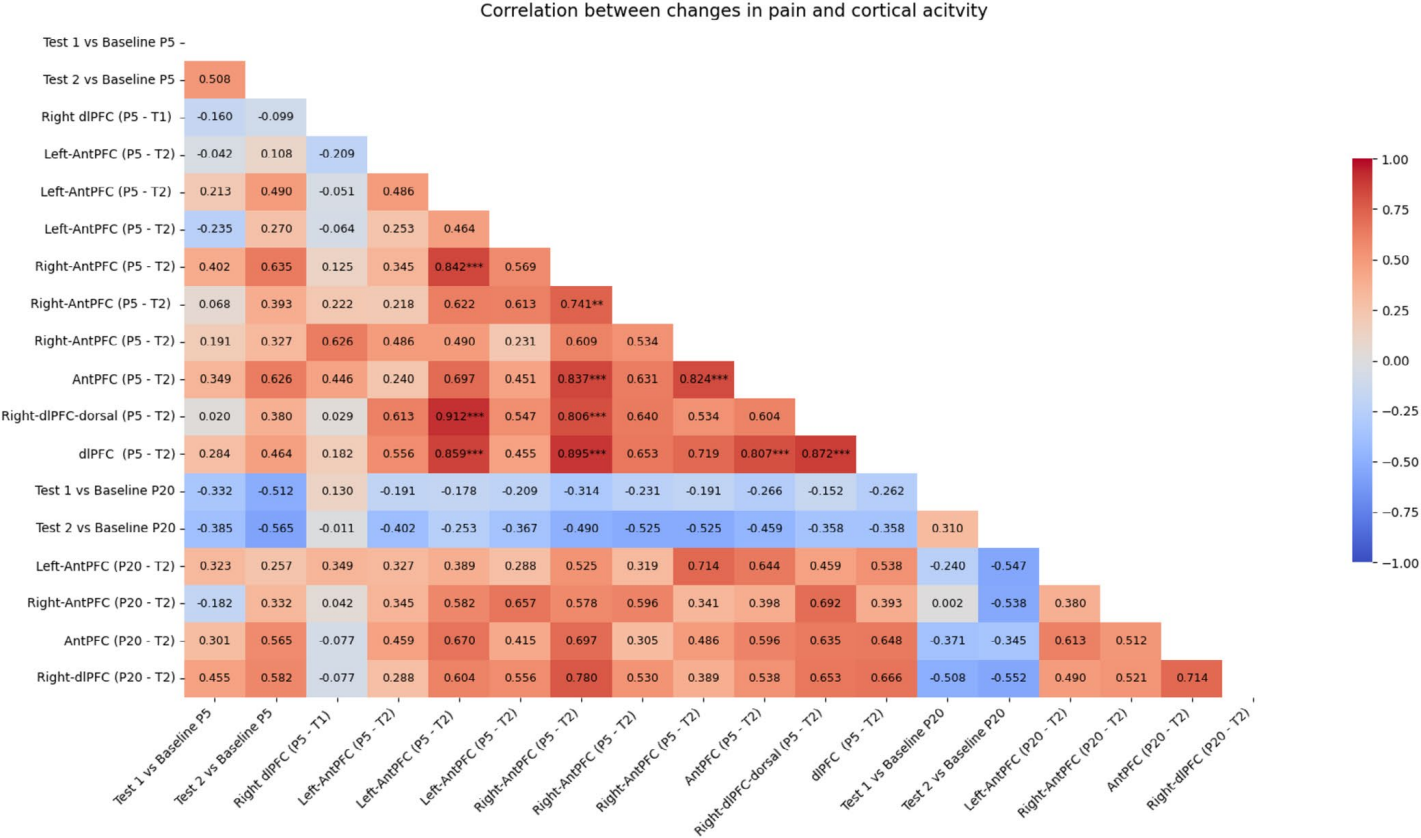
Correlation analysis between differences in pain perception and beta values (brain activity) from Baseline to Test sessions. From top to bottom, and from left to right, first the data for the P5 group at Test 1 (T1), then for the P5 group at Test 2 (T2). After data for the P20 group at T1, then at T2. *p < 0.05; **p < 0.01; ***p < 0.001.

Spearman's rho analysis was carried out to investigate whether pain perception differences between Baseline and Test sessions were associated with brain activation changes, computed as differences between Baseline and Test sessions in beta values (Figure 7). Activations in prefrontal cortical areas were associated with pain relief. No correlations were found to be significant after multiple comparison corrections for the contrast of interest. Nevertheless, strong positive correlations between cortical areas were found to be significant. For the P5 group, the left‐AntPFC (channel 19) varies its activity with the dlPFC (channel 25—*ρ* = 0.859, *p* = 0.00006), the right‐dlPFC‐dorsal (channel 40—*ρ* = 0.912, *p* = 0.0000), and the right‐AntPFC (channel 48—*ρ* = 0.842, *p* = 0.0002). The right‐AntPFC (channel 48) varies its activity with the right‐AntPFC (channel 49—*ρ* = 0.741, *p* = 0.0035), the AntPFC (channel 52—*ρ* = 0.837, *p* = 0.0003), the right‐dlPFC‐dorsal (channel 40—*ρ* = 0.806, *p* = 0.0008), and with the dlPFC (channel 25—*ρ* = 0.895, *p* = 0.0000). The right‐AntPFC (channel 50) correlates with the AntPFC (channel 52—*ρ* = 0.824, *p* = 0.0005). The AntPFC (channel 50) correlates with the dlPFC (channel 25—*ρ* = 0.807, *p* = 0.0008), and the right‐dlPFC‐dorsal (channel 20) correlates with the dlPFC (channel 25—*ρ* = 0.872, *p* = 0.0000).

## 5 Discussion

The present study's aim was to exploratively investigate cortical regions' activation and deactivation patterns following specific verbal suggestions, implementing fNIRS technology in an already tested paradigm. Behavioural results replicated previous findings in the time domain, with a reduction in pain levels at the expected timings (Camerone et al. 2022; Camerone, Piedimonte, et al. 2021). Here, it is newly demonstrated that the placebo effect can be *switched on* at the exact time point suggested by the experimenters to participants. The placebo group, which was induced to believe the cream was effective after 5 min, experienced the effect shortly and maintained it at different sessions, while the placebo group, which was induced to believe the cream was effective after 20 min, experienced the placebo effect only at the suggested test session. Interestingly, the P20 group received the cream at the same time as the P5 group and was believed to be more effective than the NE group (control). Therefore, participants had high expectations on the efficacy and already had the cream on their arm, but, nevertheless, did not experience placebo analgesia until the verbal suggestion previously received allowed them to. This finding stresses the relevance for operators in clinical care settings to provide direct and clear information on the treatment's efficacy onset. If the placebo effect can follow the drug's delayed pharmacological responses to the condition's symptoms, patients' outcomes could be improved (Carlino et al. 2012). On the contrary, if pharmacological effects take on but patients have already lost hope in the treatment's efficacy and trust in the clinician's instruction, losing benefits from the placebo effects, the overall outcome could be weakened, as demonstrated by open‐hidden experiments on drug effectiveness (Benedetti et al. 2003). Additionally, we found a strong correlation as higher expectations about the cream's effectiveness were associated with greater pain reduction at both T1 and T2 in the P5 group and at T2 for the P20 group. To further investigate this association, we carried out moderation analyses, which have not found any moderation effect operated by expectations on pain reduction. The debate on whether expectations directly influence reported pain sensation is still open; these findings align with Colloca et al. (2020), who found that expectations in patients correlated with, but did not mediate, the placebo effect, which was instead driven by prior experiences. Similarly, open‐label placebo studies (e.g., Emadi Andani et al. 2024; Schaefer et al. 2018) show that analgesia can occur even when expectations are minimal. Further investigations are needed to explain the causal role of subjective expectations on the placebo effect expression: it is possible that more affective and relational components, such as trust and hope, may correlate more strongly with pain relief than expectations about treatment efficacy.

Investigating if this effect translates also to cortical modulation, we exploited 46‐channel fNIRS, covering the whole prefrontal cortical areas, already known to play a crucial role in the expression of the placebo effect (Crawford et al. 2023; Wager et al. 2004). We chose not to select a priori ROIs relevant to prefrontal areas to better represent the unknown nature of the placebo effect modulated by time information. fNIRS analysis showed a significant increase in activity of the right‐dorsolateral prefrontal cortex (right‐dlPFC) between the early Test sessions, after 10 min (T1), compared to the Baseline session, only in participants who received verbal suggestions of an early pain reduction. At T2, tightly mimicking the behavioural results, a significantly higher activity in the right‐dlPFC was observed in participants of both groups (P5 and P20). Therefore, changes in reported pain perception not only happen on a behavioural level, but it seems that brain activity follows verbal suggestion of relief at the expected time points. Moreover, during T2, a significantly higher activation of the left postcentral gyrus as well as widespread activation of the anterior part of the prefrontal cortex were also observed in both placebo groups. These last activations might be interpreted as the encoding of reward anticipation and time perception itself. Indeed, different studies have shown how the cortical and subcortical representation of time perception partially overlaps with areas generally involved with predictions of future rewards. These areas involve both subcortical areas, such as the basal ganglia (Coull et al. 2011), as well as cortical areas such as the prefrontal and parietal cortices, and the postcentral gyrus (Üstün et al. 2017; Apaydın et al. 2018). Exploring correlational findings between changes in pain perception and brain activity in prefrontal cortical areas, no significant results were found; instead, several strong and significant intra‐prefrontal associations emerged, particularly in the P5 group. This picture reflects functional connectivity patterns that may be associated with top‐down modulation of pain during placebo analgesia, supporting the role of prefrontal networks in this phenomenon.

Findings within our paradigm seem to integrate brain regions involved in the placebo effect with ones involved in the encoding of time and reward, showing a complex fronto‐parietal network present after delivering painful stimuli in the placebo groups. Interestingly, future work should focus on evaluating the time domain of the placebo effect, overcoming event‐related block paradigms, which, albeit significant, do not encompass the complexity of the phenomenon studied. Our results on the correlation between different frontal channel demand for further functional connectivity analysis and paradigms structured to investigate brain activity shifts in real time can lead to more ecological paradigms, carried out with fNIRS to provide more comfort and adaptability compared to fMRI. On the other hand, fNIRS, while balancing temporal and spatial resolution, focuses on cortical areas and lacks subcortical information, which is crucial for time perception and reward systems. Although significant results were observed and power analysis ensured an adequate sample size, increasing participants would lessen physiological variability. Our findings are exploratory and the first attempt to elucidate brain activity regarding the onset of placebo effects. Moreover, coming from study results, future work could consider evaluating expectations throughout the whole experiment, and not a one‐time only once before the Test sessions, together with different constructs as previously mentioned, to investigate why it seems that expectations do not modulate changes in pain perception. Moreover, investigating whether the placebo effect may undergo an induced *switch‐off* by verbal suggestions would be a promising continuation of this work and a necessary step toward a comprehensive understanding of the temporal dynamics of placebo analgesia.

## Author Contributions

Conceptualisation: A.P., E.C., V.V. Methodology: A.P., E.C. Formal analysis: V.V., A.P. Investigation: V.V., F.C., E.M.C., F.P. Writing – original draft: V.V., A.P., E.C. Writing – review and editing: V.V., E.M.C., F.C., F.P. Supervision: A.P., E.C., E.M.C. Project administration and funding acquisition: E.C. All authors have seen and approved the final version of the manuscript submitted; the article is the authors' original work, is not under consideration or has not received prior publication elsewhere.

## Conflicts of Interest

The authors declare no conflicts of interest.

## Data Availability

The data supporting this work are available from the corresponding author upon reasonable request.
